# Association between tumor architecture derived from generalized Q-space MRI and survival in glioblastoma

**DOI:** 10.18632/oncotarget.16296

**Published:** 2017-03-16

**Authors:** Erik N. Taylor, Yao Ding, Shan Zhu, Eric Cheah, Phillip Alexander, Leon Lin, George E. Aninwene, Matthew P. Hoffman, Anita Mahajan, Abdallah S.R. Mohamed, Nathan McDannold, Clifton D. Fuller, Clark C. Chen, Richard J. Gilbert

**Affiliations:** ^1^ Chemistry and Chemical Biology, Northeastern University, Boston, MA, USA; ^2^ Radiation Oncology, University of Texas, MD Anderson Cancer Center, Houston, TX, USA; ^3^ Department of Radiology, Brigham and Women’s Hospital, Boston, MA, USA; ^4^ Department of Engineering Science, University of Oxford, Oxford, UK; ^5^ Center for Theoretical and Applied Neuro-Oncology and Department of Neurosurgery, University of California, San Diego, CA, USA

**Keywords:** glioma, diffusion weighted MRI, cancer biomarkers

## Abstract

While it is recognized that the overall resistance of glioblastoma to treatment may be related to intra-tumor patterns of structural heterogeneity, imaging methods to assess such patterns remain rudimentary. Methods: We utilized a generalized Q-space imaging (GQI) algorithm to analyze magnetic resonance imaging (MRI) derived from a rodent model of glioblastoma and 2 clinical datasets to correlate GQI, histology, and survival. Results: In a rodent glioblastoma model, GQI demonstrated a poorly coherent core region, consisting of diffusion tracts <5 mm, surrounded by a shell of highly coherent diffusion tracts, 6-25 mm. Histologically, the core region possessed a high degree of necrosis, whereas the shell consisted of organized sheets of anaplastic cells with elevated mitotic index. These attributes define tumor architecture as the macroscopic organization of variably aligned tumor cells. Applied to MRI data from The Cancer Imaging Atlas (TCGA), the core-shell diffusion tract-length ratio (c/s ratio) correlated linearly with necrosis, which, in turn, was inversely associated with survival (*p* = 0.00002). We confirmed in an independent cohort of patients (*n* = 62) that the c/s ratio correlated inversely with survival (*p* = 0.0004). Conclusions: The analysis of MR images by GQI affords insight into tumor architectural patterns in glioblastoma that correlate with biological heterogeneity and clinical outcome.

## INTRODUCTION

Glioblastoma is the most common form of adult primary malignant brain tumor [1]. It remains one of the deadliest of human cancers, with median survival of approximately 14 months [2]. One of the most notable features of glioblastoma is the significant degree of regional biological heterogeneity apparent on pathological inspection [3–6]. The tumor was originally termed glioblastoma multiforme because of the variegated appearance of white, hyper-cellular regions interwoven with regions of yellow necrotic tissues, zones of hemorrhagic tissues, and cystic areas [3]. We applied herein an image analysis algorithm, termed generalized Q-space imaging (GQI), to determine whether regional patterns of glioblastoma tissue organization could be derived non-invasively, and whether such patterns are substantially related to the underlying tumor biology and clinical outcome. In so doing, we defined a novel macroscopic feature of glioblastoma, that is, intra-tumor variability of alignment that appears to correlate with regional differences of necrosis and anticipates clinical performance.

Diffusion-weighted magnetic resonance imaging (DW-MRI) has been employed to assess various tumor features and predict clinical outcome [7–12]. Current methods of DW-MRI analysis are generally based on diffusion tensor imaging (DTI) algorithms that depict tumor organization through net cellular orientation (fractional anisotropy, FA) [13] or regional cellularity (apparent diffusion coefficient, ADC) [10, 14]. Enhancing the utility of these methods with tractography DTI has been used to predict surgical outcome from the patterns exhibited by co-aligned neural fibers [15, 16] and to assess brain connectivity [17]. GQI is a novel diffusion-weighted method that derives complex intra- and inter-voxel fiber alignment in tissue, differing from DTI principally in that it assumes neither a Gaussian distribution nor intra-voxel signal uniformity. GQI systematically interrogates diffusion space, representing the probability of diffusional motion in multiple directions for each voxel as a unique probability distribution function (PDF). The directions of maximal diffusion per voxel may then be linked by tractography [18, 19] to generate maps of distributed tracts throughout the tissue. Architectural features that are regionally heterogeneous, typified, for example, by intravoxel crossing [18] or local edema [20], should be optimally visualized by GQI. We hypothesized in the current study that regional structural differences of tumor architecture in glioblastoma could be depicted with GQI, and that such attributes relate to underlying tumor biology and clinical outcome. Our results indicate that GQI detects unique intra-tumor structural features in rodent and human glioblastoma that correlate both with intra-tumor biological heterogeneity and overall survival.

## RESULTS

### Rodent model of glioblastoma tumor architecture

**Figure 1:**
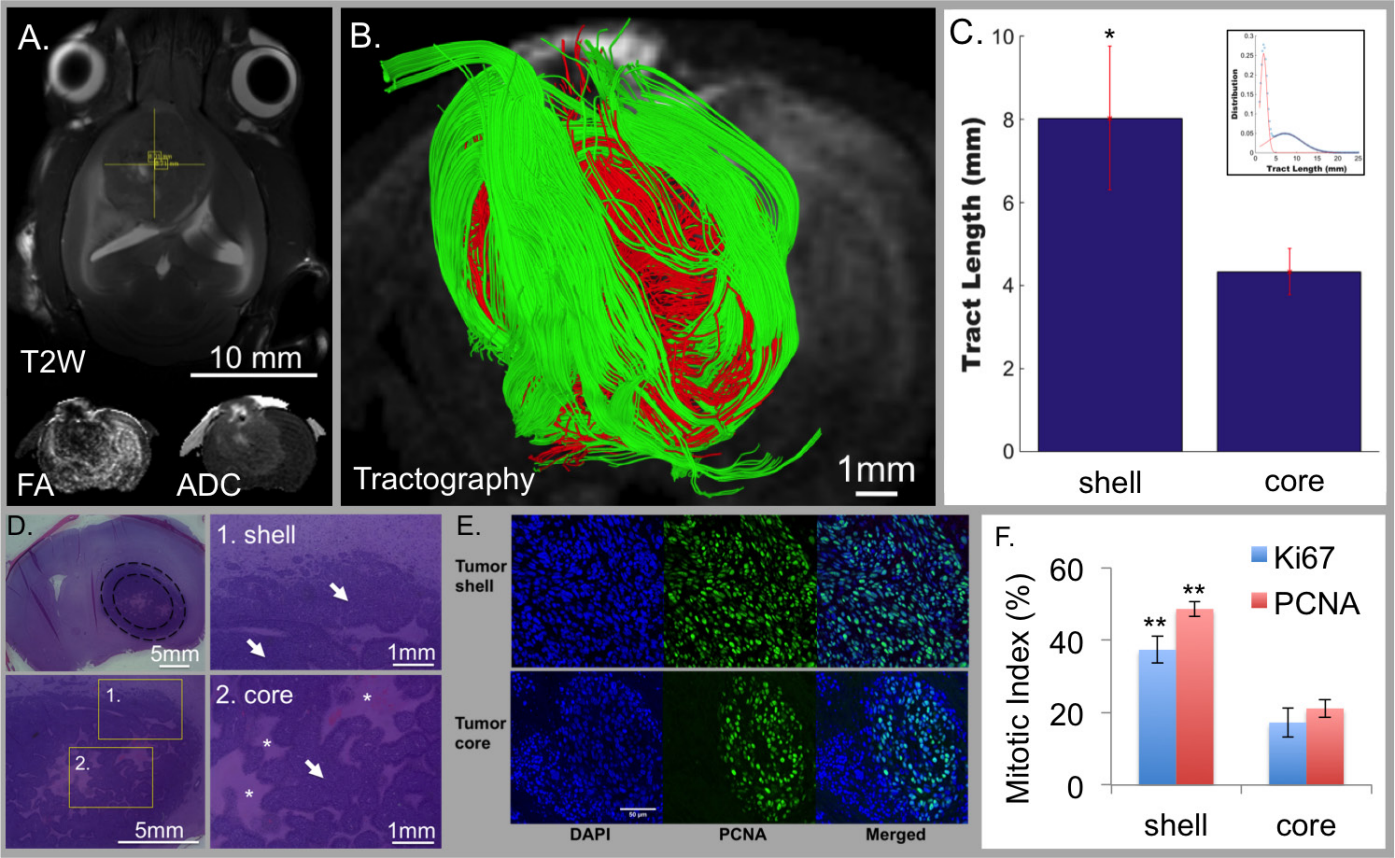
Tumor architecture obtained through GQI tractography in rats injected with F98 glioblastoma cells ( ***N*** = 3). Conventional MRI demonstrated tumor location with T2-weighting (T2W; axial view in **A**.) and diffusion-weighting (DW; inset in coronal view) with fractional anisotropy (FA) and apparent diffusion coefficient (ADC). GQI depicted cellular diffusion orientation-coherence (coronal view; **B**.) with core (red; 1-5 mm tracts) and shell (green; 6-25 mm tracts) employing a b-value of 1200 s/mm^2^ and 512 gradient directions. **C**. Quantification of tract-length in rat tumors (1-25 mm tract-length) using spatially distinct regions demonstrated significantly greater cellular alignment at the shell of 8.0316+/− 1.7275 mm versus core of 4.3299+/− 0.555 mm (*p* < 0.01); inset, statistically distinct aligned cellular populations from bi-Gaussian distribution, with short and long tract-lengths at mean values of 1.9922 mm and 7.1214 mm, and respective mixing proportions of 0.502814 and 0.497186. **D**. H&E histology of coronal slices; regions are inset at 1) shell and 2) core. The shell-region consisted of sheet-like structures, while the core was disorganized with a large degree of central necrosis. White arrows indicate tumor tissue and white asterisks indicate regions of necrosis. **E**. Differential mitotic activity of cancer cells at the shell and core with DAPI (nuclear stain; blue) and PCNA (mitotic activity; green). **F**. Mitotic index with PCNA and Ki67 demonstrated that glioblastoma cells in the shell regions are highly mitotic proliferative tissues compared to the core regions (*p* < 0.001). Scale bars represented are in A. 10 mm, B. 1mm, D. 5mm or 1 mm, and in E. 50 μm, consistent across the panel.

We generated a brain tumor model through injection of F98 glioblastoma cells into rats (Figure 1). Twenty-six days after tumor injection, the rats were studied with T2-weighted (T2W) MRI or DTI derived fractional anisotropy (FA) and apparent diffusion coefficient (ADC) (Figure 1A). When GQI tractography was applied to DW-MRI, unique regional differences were visualized (Figure 1B). GQI tractography demonstrated millimeter-range tracts (6-25 mm tracts; green), consisting of aligned glioblastoma cells (Figure 1B) in a peripheral zone (shell) encapsulating a central region (core) of short-range orientation coherence (1-5 mm tracts; red). Secondary quantification of tract-length from tractography maps by axial-defined radial volumes in tumors (Figure 1C) and independent histogram analysis (inset in 1C) validated that the core and shell tumor sub-regions were distinguished by short or long tract-length. Core-shell tract-length architecture correlated anatomically with two distinct histological regions, namely an organized peripheral zone, possessing sheets of aligned glioblastoma cells, and a disorganized central zone, possessing poorly aligned cells and necrosis (Figure 1D). The mitotic index of glioblastoma cells was quantified at the tumor shell and core regions using antibodies to proliferating cell nuclear antigen (PCNA) or Ki67 combined with DAPI (PCNA staining with DAPI shown in Figure 1E). Quantification indicated the presence of proliferative tissues at the shell region, with a significantly greater mitotic index (*P* value < 0.001) at a fold increase of 2.30 and 2.16 compared to the core region with PCNA or Ki67, respectively (Figure 1F).

### GQI analysis of clinical glioblastoma MRI derived from TCIA dataset

**Figure 2:**
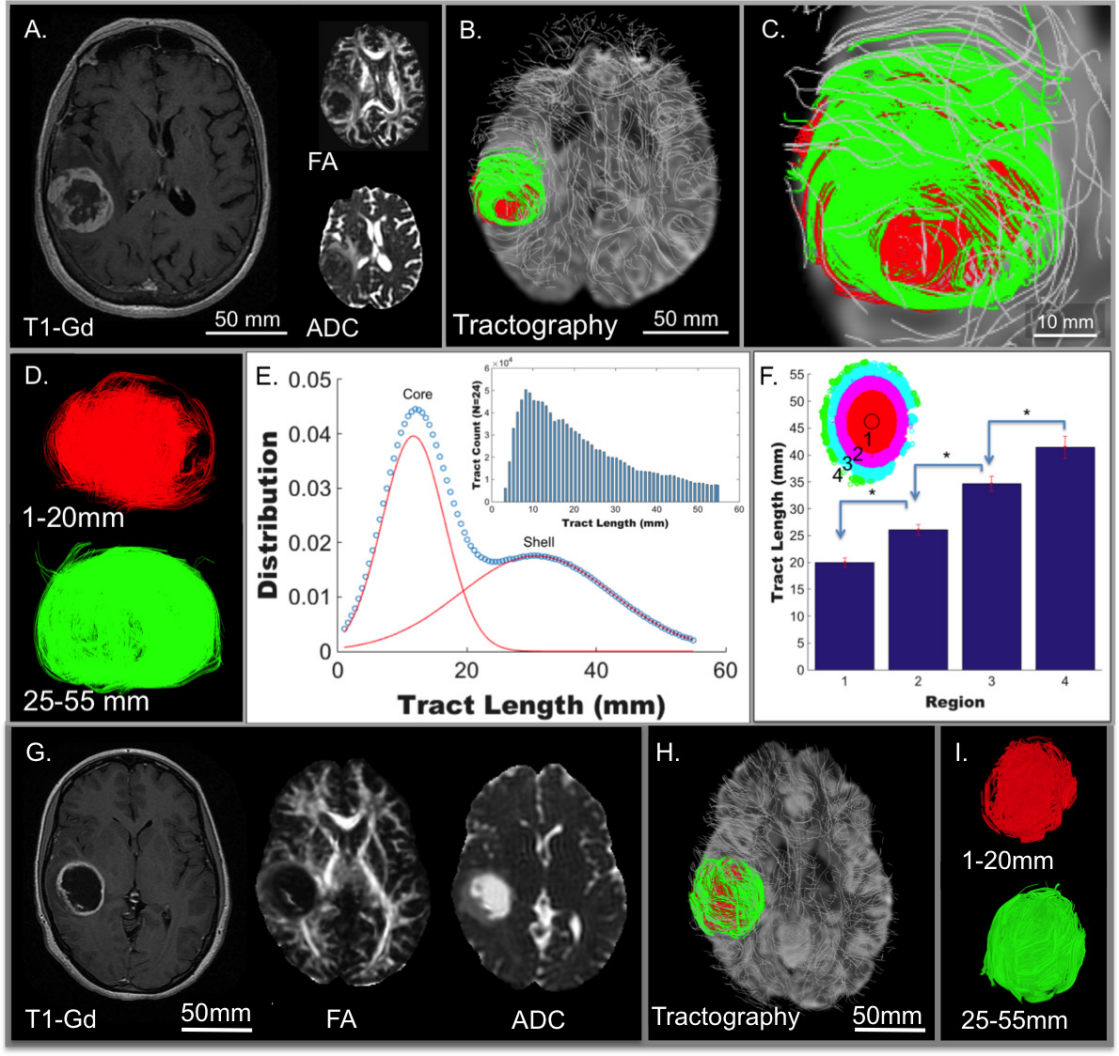
Tumor architecture derived in patients from The Cancer Genome Atlas (TCGA) glioblastoma study ( ***N*** = 24). From this dataset, twenty-four patients with pre-treatment scans were identified in The Cancer Imaging Archive (TCIA) as derived from Henry Ford Hospital (19 subjects) and Case Western (5 subjects). A representative 78-year-old patient (TCGA-06-5412) is shown, demonstrating clear tumor borders when scanned employing conventional MRI including T1W post-gadolinium MRI (T1-Gd; axial view in **A**.) and diffusion-weighting (DW; axial view inset in A) with fractional anisotropy (FA) and apparent diffusion coefficient (ADC). The DW pulse sequence in the same patient was analyzed by GQI (axial view in **B**.; expanded in **C**.) with background neural tracts displayed in silver. A tract-length filter assessed regional cellular diffusion orientation coherence in glioblastoma, as shown in **D**., with core (red; 1-20 mm tracts) and shell (green; 25-55 mm tracts) tumor architecture. **E**. Two statistically distinct aligned cellular populations were demonstrated, with a bi-Gaussian distribution of tract-length in 24 patients, mean tract-lengths of 11.6937 mm and 30.6898 mm, and with mixing proportions of 0.481503 and 0.518497, respectively. **F**. The same 24 patients with quantification of tract-length at spatially distinct concentric regions grouped radially from the axial-orientation at the tumor center, defined from the average of tract points, to the edge of the tumors, consecutively labeled into four regions (*P* < 0.005 for each compared region). Representative patient (TCGA-06-2570) with glioblastoma scanned employing conventional MRI (in **G**.) and analyzed with GQI for tumor architecture (**H**.; expanded in **I**.) demonstrating high core-shell tract-length ratio (c/s ratio) in contrast to low c/s ratio in TCGA-06-5412 in A-D. Tract-length filters were applied, with core in red (1-20 mm tracts) and shell in green (25-55 mm tracts). Substantial overlap of the two regions was observed in this patient, and was representative of the difference between the high and low c/s ratio patients.

To demonstrate the utility of GQI analysis in glioblastoma patients, we identified 24 patients from The Cancer Imaging Archive (TCIA), where multi-direction DW-MRI employing imaging parameters sufficient for GQI analysis were employed (b-value of 1000-1200 s/mm^2^ and gradient directions of 25-37). A representative 78-year-old patient (TCGA-06-5412; patient TCGA #1) is shown (Figure 2), in which the tumor is localized in an axial view with T1-weighted post-gadolinium MRI (T1-Gd), FA, and ADC (Figure 2A). In every case analyzed, GQI demonstrated regional heterogeneity as determined by tractography maps. GQI demonstrated tumor sub-regions defined spatially by orientation with either short (1-20 mm tracts; red) or long (25-55 mm tracts; green) tract-length, designated as core and shell, respectively (Figure 2B-2D). Secondary quantification of heterogeneous tumor orientation coherence was demonstrated in a histogram of tract-length from the group of glioblastoma patients (*N* = 24; statistical bi-Gaussian distribution shown in Figure 2E with histogram inset). Tract-length was independently derived within spatially distinct concentric rings (located in the axial plain) defined from the tumor center and then selected radially towards the tumor edge, whereby a significant difference in tract-length was observed over the clinical cohort and between each consecutive region (Figure 2F; inset is an example of the regions measured from patient TCGA #1). As was observed in the rat glioblastoma model, regional architecture was not detected using conventional DTI analysis (ADC or FA) or by DTI with tractography (Supplemental Figure S1).

### Association between glioblastoma c/s ratio, necrosis, and overall survival

**Figure 3:**
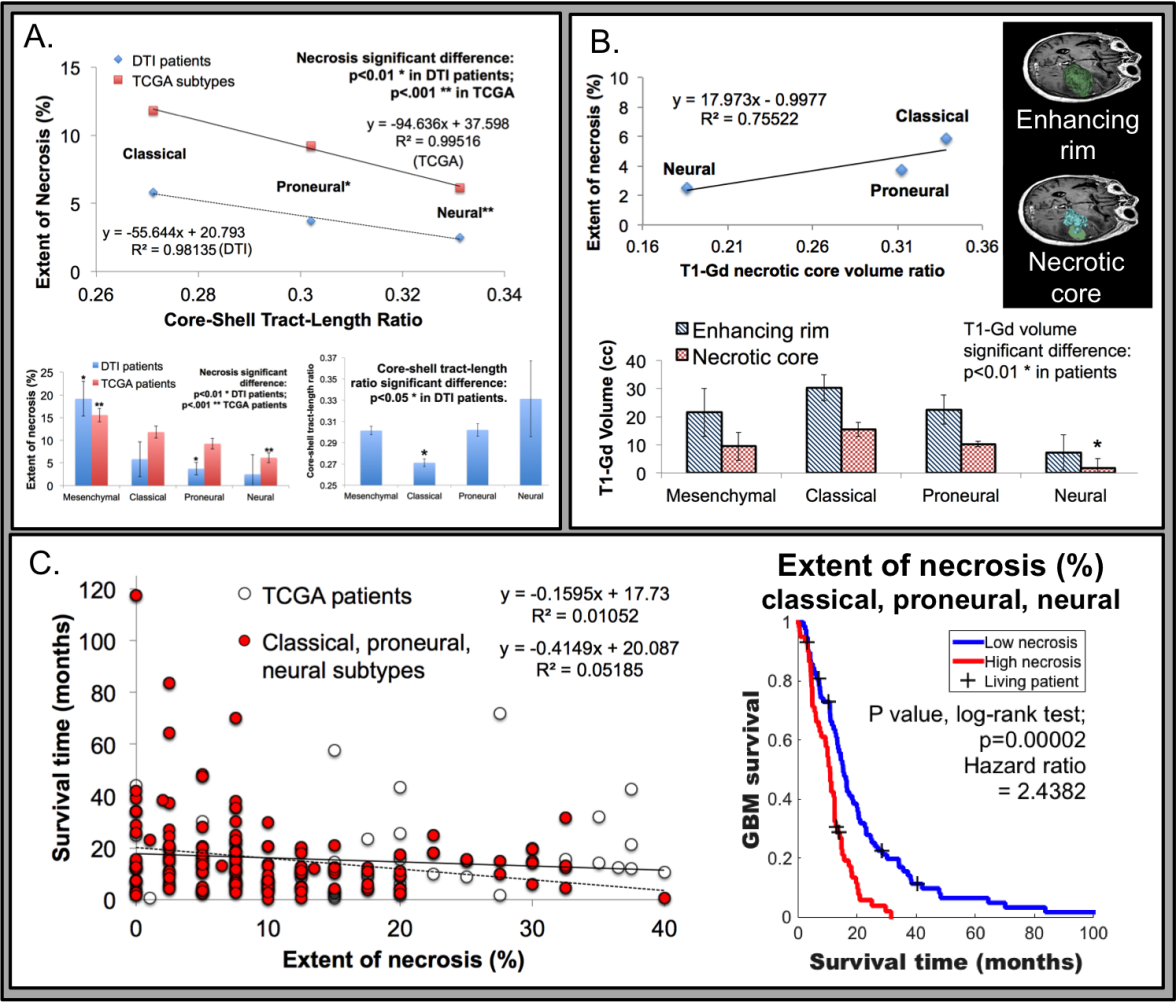
Association of tumor necrosis relative to genomic subtypes from The Cancer Genome Atlas (TCGA) and MRI derived metrics **A**. Tissue necrosis, derived from histology, was inversely related to the c/s ratio, with a linear relationship observed (R^2^ value 0.9814 in DTI patients; or 0.9951 with TCGA patients; *N* = 138) in the classical, pro-neural, and neural subtypes. Inset bottom left, the extent of necrosis correlated to tumor subtype, with the mesenchymal subtype (NF1 mutations or loss with mesenchymal markers) displaying a significantly higher degree of necrosis compared to all others (*p* < 0.01 in 24 DTI patients and *p* < 0.001 in 196 TCGA patients as derived by Verhaak et al). The neural subtype (neuron-like glioblastoma genotype) was significantly correlated with the least amount of necrosis (*p* < 0.001 from TCGA). Inset bottom right, the relationship between all glioblastoma subtypes and the GQI derived c/s ratio, with the classical (*EGFR* amplification; *N* = 3) subtype demonstrating the lowest c/s ratio (*p* < 0.05), compared to all other subtypes including mesenchymal (*N* = 9), pro-neural (alterations of *PDGFRA* and point mutations in *IDH1*; *N* = 9), and neural (*N* = 3). **B**. Histologically derived extent of tissue necrosis was correlated to T1-Gd derived necrotic core to enhancing rim volume ratio (R^2^ value 0.7552 in DTI patients; representative analysis of T1-Gd patient data is inset top right). Inset bottom, the neural subtype had the least necrotic core volume (*p* < 0.01 from DTI patient data), while other subtypes were not distinguished. **C**. The relationship between survival time and tumor necrosis in TCGA patients was inversely correlated (*N* = 196), with significance achieved in the classical, pro-neural, and neural subtypes (univariate analysis; *p* < 0.01; *N* = 138). Inset right, Kaplan-Meier survival analysis with the log-rank test demonstrated a significant survival relationship with extent of necrosis in classical, pro-neural, and neural subtypes (78 patients versus 60 with extent of necrosis 0-7.5% or 10-40%, respectively). Censoring events are indicated with a + tick mark and labeled as living patients.

To elucidate the role that macroscopic heterogeneous tumor organization plays in patient specific tumor properties and outcomes, the core-to-shell tract-length ratio (c/s ratio) was established (Figure 3). In our analysis of these 24 TCIA patients, we noted significant differences of the c/s ratio among the various patients (Figure 2A-2D and 2G–2I illustrate patients with low vs. high c/s ratio, respectively). To determine whether the c/s ratio is associated with varying degrees of regional tumor necrosis, we examined histological data for 15 of these 24 patients. Consistent with our rodent model dataset, we found that the patients with higher c/s ratios were associated with decreased necrosis, whereas those with lower c/s ratios exhibited increased necrosis (Figure 3A). We validated the aforementioned correlation with necrosis in 15 selected TCIA patients using the ratio of the tumor-enhancing rim and non-enhancing core on T1-Gd imaging as an imaging proxy for the extent of necrosis [21, 22]. Significantly, within this 15 patient cohort, the c/s ratio linearly associated with the extent of tissue T1-Gd necrosis for all three glioblastoma subtypes (Figure 3B).

To determine whether the association between c/s ratio and necrosis persists after controlling for the existence of transcriptional subtypes of glioblastoma [23–25], we examined histological and transcriptional subtype data for an additional 196 patients from The Cancer Genome Atlas glioblastoma dataset (Figure 3C). When stratified by subtype, the mesenchymal subtype was associated with significantly greater tissue sample extent of necrosis compared to all other subtypes. Moreover, there is little variation between mesenchymal glioblastomas in terms of the extent of necrosis, rendering correlative analysis between c/s ratio and extent of necrosis not feasible. In contrast, the proneural, neural, and classical subtypes exhibit varied extent of tumor necrosis, with the classical subtype with the highest level of necrosis and the neural subtype with the least amount of necrosis (inset in Figure 3A; *N* = 196 total patients from TCGA; *p* < 0.001). Moreover, for the remaining three subtypes, we observed consistent relationship between extent of necrosis and overall survival (Figure 3C; univariate analysis; *p* < 0.01; *N* = 138 patients). When necrosis is controlled for by multivariate analysis in classical, pro-neural, and neural subtypes, the survival correlation was independent of subtype (*p* < 0.01 for necrosis vs. p>0.05 for each subtype). The majority of these TCGA patients were male (61.88%), and age or KPS significantly correlated to survival. Necrosis was shown to be independent of these clinical features in multivariate analysis and remained significantly correlated to TCGA patient survival (*p* < 0.01).

### Validation of the association between c/s ratio and overall survival in an independent dataset

**Figure 4:**
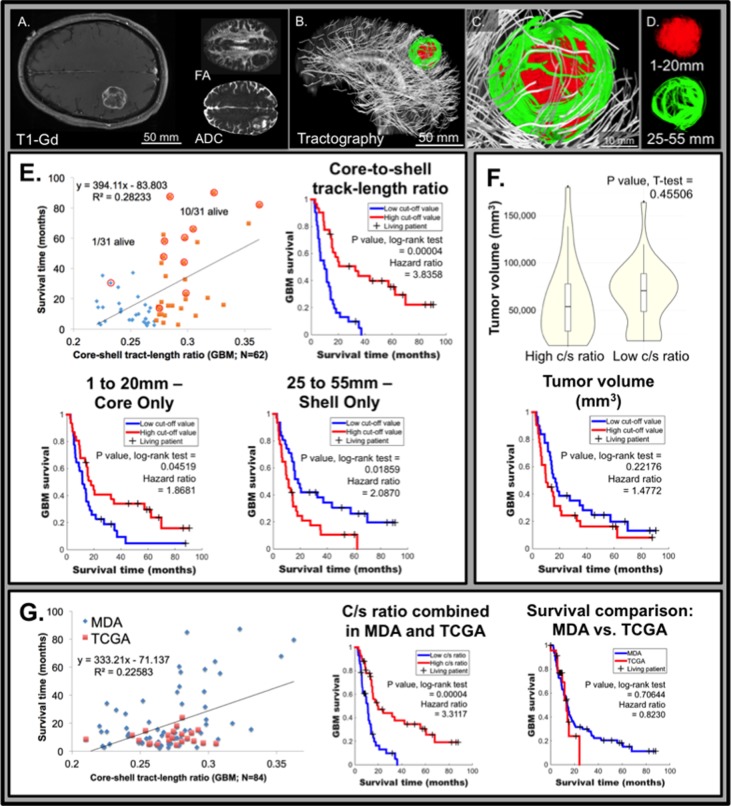
Relative glioblastoma tumor architecture determined from GQI tractography in the University of Texas, MD Anderson Cancer Center (MDA) patient dataset correlates with survival ( ***N*** = 62). Representative patient MDA #1 with glioblastoma, demonstrating clear tumor borders, as scanned employing conventional MRI including T1W post-gadolinium (T1-Gd; axial view; **A**.) and diffusion-weighting (DW; axial view inset in A) with fractional anisotropy (FA) and apparent diffusion coefficient (ADC). The diffusion weighted pulse sequence in the same patient was analyzed by GQI (sagittal view in **B**.; expanded in **C**.) with background neural tracts displayed in silver, employing a b-value of 1200 s/mm^2^ and 27 gradient directions. Tract-length filters assessed regional cellular diffusion orientation-coherence in glioblastoma, as shown in **D**., with core (red; 1-20 mm tracts) and shell (green; 25-55 mm tracts) tumor architecture. **E**. Glioblastoma patient survival versus core-shell tract-length ratio (c/s ratio) displaying a linear correlation across MDA patients (*N* = 62). The 50% point of demarcation was established for comparison of high and low c/s ratio population proportions, and patients living at the time of analysis are circled (censoring events; 1/31 living for the low c/s ratio and 10/31 living for the high c/s ratio). Inset top right, Kaplan-Meier survival analysis demonstrated a significant difference between the high and low c/s ratio populations, at 50% threshold, with a p value = 0.00004 by the log-rank test and the hazard ratio between the groups was calculated to be 3.8358. Inset bottom left, analysis of the core independently (1 to 20 mm tract-length filter applied) demonstrated significant difference of p value = 0.04519 by the log-rank test, at 50% threshold, with a hazard ratio of 1.8681. Inset bottom right, analysis of the shell independently (25 to 55 mm tract-length filter applied), where a significant difference of p value = 0.01859 by the log-rank test was demonstrated, at 50% threshold, and with a hazard ratio of 2.0870. **F**. Tumor volume was statistically equivalent between the low and high c/s ratio patients, shown by violin plots with a p value = 0.45506. Inset bottom, tumor volume as an independent factor did not predict survival difference at 50% threshold, with a p value = 0.22176 by the log-rank test. **G**. Glioblastoma patient survival versus GQI derived c/s ratio displaying a linear correlation normalized to the mean across all patients (MDA plus TCGA; *N* = 86). Inset middle, a significant difference between the high and low c/s ratio populations in the combined MDA and TCGA dataset was found, at 50% threshold, with a p value = 0.00004 by the log-rank test and the hazard ratio between the groups was calculated to be 3.3117. Inset right, survival in the TCGA and MDA datasets were statistically equivalent, with p value = 0.70644 by the log-rank test.

We studied the association between the c/s ratio, necrosis, and survival in an independent cohort of pre-operative MRI obtained from 62 glioblastoma patients (separate from the TCIA dataset). The cohort was annotated in terms of key clinical characteristics including age, Karnofsky Performance Status (KPS), overall survival, and molecular profiling of the tumor specimens. A representative 61-year-old patient is shown in Figure 4, with glioblastoma location demonstrated by T1-Gd, FA, and ADC (Figure 4A). Similar to the rodent model and TCGA/TCIA human data, GQI demonstrated spatial heterogeneity characterized by distinct core and shell tumor sub-regions (Figure 4B-4D). The presence of variably aligned cancer-cells was depicted through generation of a bi-Gaussian histogram and through definition of spatially distinct concentric rings (Supplemental Figure S2). Confirming our hypothesis, the GQI derived c/s ratio was associated through univariate analysis (*p* = 0.000009) with overall survival in this cohort (Figure 4E). This association persisted after controlling for clinical variables (through multivariate analysis) known to influence survival, include age and KPS. To control for potential effects related to tumor volume or treatment regimens, we divided the cohort into two groups based on median c/s ratio, and observed that tumor volume and treatment regimen were indistinguishable between these two groups. Survival in the high c/s group was significantly better than the low c/s group (Figure 4E). Representative patient MDA #1 from the low c/s ratio group is shown in Figure 4A-4D and representative patient MDA #2 from the high c/s ratio group is shown in Figure S2C-E. To consider the potential contribution of surgical resection to the observed survival outcomes, we analyzed the eight patients who underwent tumor biopsy without resection (Supplemental Figure S3). In these patients, the presence of a low c/s ratio corresponded to a significantly worse prognosis (*N* = 4; hazard ratio of 14.1182) relative to the high c/s ratio patients, suggesting that the association between c/s ratio and overall survival is independent of surgical intervention. To consider the potential influence of the isocitrate dehydrogenase (*IDH*) on the association between c/s ratio and overall survival, we screened 42 patients with sufficient clinical specimen for *IDH* mutation. Of these patients, Six (9.6%) harbored *IDH* mutations. We found that the c/s ratio remained closely associated with survival in a statistical model that accounted for *IDH* mutation status (c/s ratio *p* = 0.0009; *IDH* wild-type glioblastoma).

## DISCUSSION

Given the high mortality associated with glioblastoma, there is substantial need for non-invasive methods to classify the biological behavior of these tumors. We have developed such an approach, based on principals of generalized Q-space imaging (GQI) with tractography, a method that derives macroscopic features of tumor organization from directional variations of proton diffusion *in vivo* [18, 19]. Through this approach we demonstrated that the diversity of tumor biological features known to exist in glioblastoma [3–6] may be represented in patients as variations in tumor architecture obtained through MRI. Moreover, we have shown that a specific tumor architectural pattern, the tumor core-shell tract-length ratio (c/s ratio), correlates with glioblastoma patient survival. Such patterns may be characteristic of tumor growth and multi-cellular organization of the tumor, [26] and reflect the complex interactions of tumor cells with their microenvironment [27]. In this context, GQI based imaging may provide a non-invasive imaging biomarker for agents that selectively target these processes.

**Figure 5:**
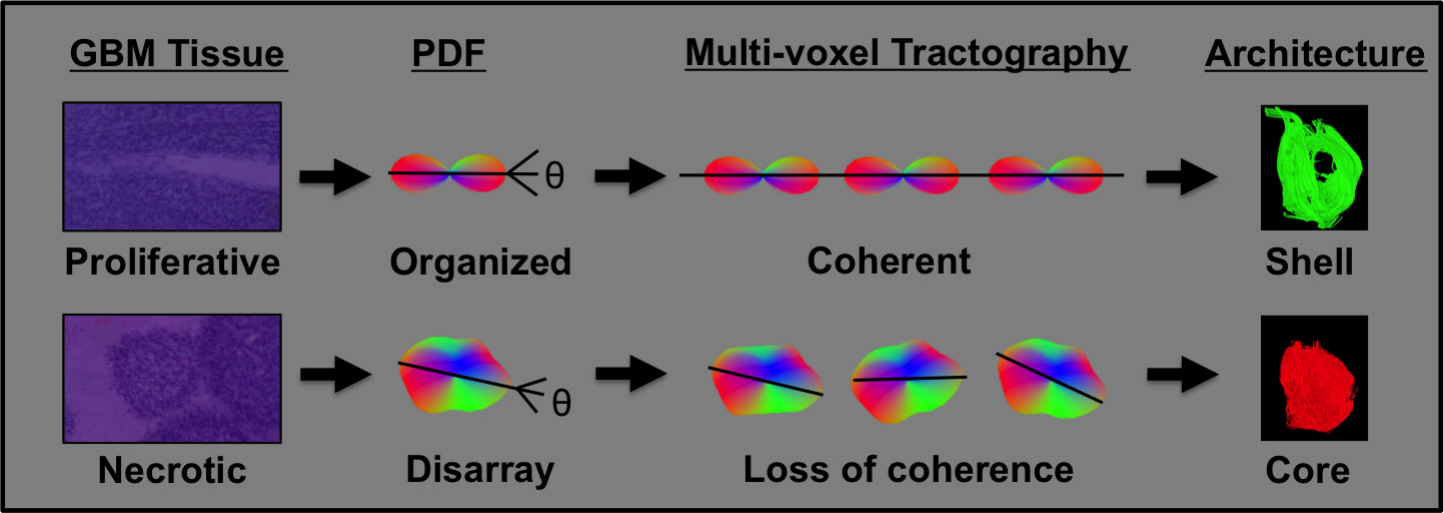
GQI defined core-shell architecture is indicative of underlying glioblastoma cancer-cell heterogeneity The outcome of GQI is a probability distribution function (pdf) per voxel that represents the underlying diffusion in terms of orientation and magnitude, and is derived from the Fourier transform of diffusion-weighted MRI data. Proliferative glioblastoma tissue in the tumor periphery contains organized cancer cells, generating a diffusion pdf that co-aligns across multiple voxels and results in the relatively long tracts present in the tumor shell. Highly necrotic glioblastoma tissue in the central region of the tumor is characterized by poorly organized cancer cells, generating altered pdf shape and a loss of coherence across multiple voxels, and resulting in relatively short tracts in the tumor core.

To our awareness, our study represents the first to apply Q-space MRI methods to the analysis of glioblastoma regional organization in rodent and human models [7, 8]. As a proof-of-principle, our analysis demonstrates the feasibility of applying GQI to clinical MRIs derived from glioblastoma patients. We define a novel quantitative metric of glioblastoma regional heterogeneity based on GQI defined diffusion tracts. We specifically observed that glioblastoma are prototypically organized into a central core consisting of short-length GQI tracts surrounded by a shell of long-length GQI tracts. Our rodent study suggest that this core region is characterized by a high degree of necrosis while the shell region is associated with sheets of organized anaplastic cells with elevated mitotic index (this principal is shown schematically in Figure 5). Moreover, glioblastoma observed in patients exhibit similar variation in the ratio of tract-length in the core and shell region. Glioblastoma with high c/s ratio are associated with minimal extent of necrosis relative to those with low c/s ratio. These results suggest utility of GQI based algorithms as a non-invasive means of assessing regional heterogeneity within glioblastoma, particularly as it relates to the extent of necrosis within the tumor. Notably, the specific imaging parameters required for GQI analysis do not significantly increase MR imaging time in patients, thus rendering clinical translation plausible.

The survival association between the c/s ratio, the extent of necrosis, and overall survival in two independent patient cohorts is important from several perspectives. First, our study independently validates a previous study suggesting an inverse correlation between extent of necrosis and overall survival in glioblastoma patients [22]. Second, our analysis indicates that the extent of necrosis differ amongst glioblastoma subtypes, with mesenchymal subtype harboring the highest extent of necrosis and neuronal subtype with minimal necrosis. Third, our study suggests that the GQI-defined c/s ratio constitutes a potential prognostic indicator, which is independent of known clinical and molecular biomarkers, including age, KPS, and *IDH* mutation. Finally, while the molecular mechanism resulting in regions of necrosis in glioblastoma remain poorly understood, it is known that cells in these regions harbor unique metabolic profiles [28], hypoxic cellular responses [27, 29, 30], and signal transduction [31]. Given the relationship between central necrosis and low oxygen tension [27, 29], the magnitude of c/s ratio could be indicative of conditions that play a role in resistance to therapy [27, 32] through the genesis of aberrant tumor vasculature, [33, 34] or the control of radiation-induced reactive oxygen species (ROS) [30], thus impacting patient survival.

We conclude that multi-direction diffusion-weighted MRI analyzed by GQI algorithms detects and quantifies regions of intra-tumoral glioblastoma heterogeneity on the basis of tumor architecture. Such regions may be characterized by patterns of diffusion co-alignment that reflect distinct differences of tumor biology. The association between intra-tumoral architecture derived from local patterns of diffusion and survival was striking. These findings suggest the potential utility of GQI-defined radiographic features as non-invasive biomarkers of clinical outcome.

## MATERIALS AND METHODS

### Generalized Q-space MRI (GQI)

GQI is a MRI method that considers the effect of pulsed field gradients and diffusion times on the characteristics of Q-space, producing a mathematical rendering of the 3D diffusion environment. This approach provides a method to analyze complex biological tissues in terms of microstructure [35]. Employing GQI, multiple gradient orientations are applied in order to evaluate signal attenuation in 3D space. The outcome is a probability distribution function (pdf) that displays diffusion in terms of a 3D orientation function multiplied by the quantitative spin density, derived from the Fourier transform of diffusion-weighted image data [18]. Optimization in GQI incorporates a nominal number of gradient directions and approximated b-values towards achieving a target angular separation, dictated by the underlying anatomy. The b-value reflects an aggregate of factors, including gradient strength and diffusion time, which contribute to diffusional sensitivity and thereby infers the capacity to probe sub-voxel diffusional barriers [18, 19].

### Rat glioblastoma model and architectural data analysis

A rat glioblastoma model was generated through the intracranial injection of F98 glioma cells. Studies were performed in 3 Fischer rats (Charles River Laboratories; ~ 250 g). Prior to tumor injection, [36] the animals were anesthetized via intraperitoneal injections of Ketamine (80 ml/kg/h) and Xylazine (10 ml/kg/h). F98 rat glioma cells (ATCC; undifferentiated malignant glioma), were grown in Minimum Essential Medium (1x) with Earle's salts, supplemented with 10% fetal bovine serum, 1% l-glutamine, 1% MEM non-essential amino acids, and 0.1% gentamicin in a 5% CO_2_ chamber held at 37°C. The biological characteristics of tumors generated by this cell line closely resemble those of human glioblastoma [8]. A 4 μl volume of cell suspension (1 × 10^5^ cells) was injected into the right caudate putamen at a depth of 3.5 mm relative to the dural surface using a 10 μl gas-tight syringe (Hamilton). Following a 26+/−4 days period of tumor growth, the animal was sacrificed via trans-cardiac perfusion with saline followed by 10% phosphate-buffered formalin while under deep anesthesia, and the brain was removed and immersed in 10% phosphate-buffered formalin [36]. Whole brains underwent diffusion-weighted MRI at 7T using a Bruker Biospec USR with a multi-shot EPI pulse sequence under the following imaging parameters: 0.2×0.2×0.4mm, b-value of 1200 s/mm^2^, and 512 gradient directions. To obtain microscopic correlation, studies were carried out in rat brains that were fixed in neutral buffered formalin, removed from the skull, embedded in paraffin, sectioned at a 6 μm thickness, and stained with hematoxylin and eosin. Histological sections of rat brain tumors were also stained with Ki67, PCNA, and DAPI, as previously described [37]. Briefly, after de-parafinization, antigen retrieval, and blocking, the slide was incubated in a moisture chamber with primary antibody for PCNA (clone PC10; Thermo Scientific), or Anti Ki-67 (rabbit; Novus Biologicals) at room temperature for 1 hour or incubated overnight at 4°C followed by secondary antibody conjugated with Alexa Fluor 594 or Alexa Fluor 488 (Life Technologies), respectively. The slide was then washed with TBST for 5 minutes and covered with DAPI mounting medium (Fisher Scientific). Confocal imaging was carried out utilizing a Zeiss LSM 710 with ZEN 10.0 software.

### Human magnetic resonance imaging

The method for determining tumor architecture employed patient data from The Cancer Imaging Archive (TCIA) derived during The Cancer Genome Atlas (TCGA) glioblastoma study. From this dataset, 24 patients with pre-treatment diffusion tensor imaging (DTI) scans were identified from two institutions Henry Ford Hospital (19 subjects) on a GE scanner with 25 gradient directions and a b-value of 1000 s/mm^2^ and from Case Western (5 subjects) on a Siemens scanner with 37 gradient directions and a b-value of 1200 s/mm^2^. For clinical validation, and to assess the significance of tumor architecture in predicting clinical outcome, magnetic resonance imaging was conducted on a 3.0 T GE Signa HDxt MRI scanner (GE Healthcare, Waukesha, WI) with an 8-channel high-resolution brain coil (GE Healthcare) at University of Texas, MD Anderson Cancer Center (MDA). Ethical approval for this research was obtained for use with anonymous and retrospective glioblastoma patient data. The pulse sequence incorporated standard single-shot echo-planar (EPI) spatial encoding, employing a voxel size of 0.86×0.86×3.5mm, 27 gradient directions plus b-zero, and a b-value of 1200 s/mm^2^.

### Tumor architecture data analysis

Tissue diffusional properties were analyzed in all instances with GQI as a representation of tumor architecture. A streamlining algorithm modified to make use of multiple diffusion directions was used to relate voxels, thus creating tracts, or visual elements that depict inter-voxel coherence. The generation of pdf with L_Δ_ = 1.25 was carried out [18] and a tracking angle of 35° was employed, as previously validated in complex organs with microscopic reconstruction [38, 39] using diffusion spectrum imaging studio (DSI Studio; http://dsi-studio.labsolver.org) [18]. MATLAB was used to find the tract distributions in tumors (1-55 mm) across the patient population, with histogram analysis and a Gaussian mixture model fit (a multivariate distribution that consists of a mixture of one or more components) from the Statistics and Machine Learning toolbox. Spatial analysis of tract-length was carried out in the axial plane (Supplemental Figure S4), first through determination of the mean tumor location in each orientation, identified from the averaging of all the points in tumor tracts at a step-size of 1 mm in humans or 0.1 mm in rat. Sampling of an axial range was carried out (20% from the center in Z plane), whereby sorting of each tract at intersection points in concentric rings was performed, with radius defined to yield four equivalent areas in each slice (equivalent only assuming circular rings defined from the max x or y range), i.e. r_i_ = R√(i/4), where R is the radius of the tumor, and r_i_ is the radius of each concentric element, and i is the number of regions. In order to sort for a particular intersecting tract location t(x_i_,y_i_), and to define an elliptical region, the radius was set as, r_t_ = √(x_i_^2^+y_i_^2^). See Supplemental MATLAB code or https://github.com/eriktaylor/ for additional details.

### Patient molecular screening and analysis

Clinical, histological, and genomic data was derived from The Cancer Genome Atlas (TCGA) glioblastoma study [24, 40]. Clinical data and extent of histological tumor necrosis was obtained (196 patients) from data available in the TCGA data portal, and correlated through previous analysis of tumor subtypes, including either classical, neural, proneural, or mesenchymal [24]. Diffusion MRI data was derived from datasets available on The Cancer Imaging Archive (TCIA), employing patients that matched with existing subtype analysis (24 newly diagnosed GBM patients were identified). Regression-based correlations were assessed in addition to Kaplan-Meier survival and multivariate analysis for necrotic features, tumor-subtypes, patient gender, and GQI derived metrics (specifics of these methods are further described below in Statistical methods and survival correlation). Tumor necrosis was determined by the Biospecimen core resource, a centralized laboratory that reviews and processes blood and tissue samples and their associated data for The Cancer Genome Atlas (TCGA) Research Network. In brief, frozen tumor sections were examined at the top and bottom, with histology microscopically assessed at multiple fields of view. For inclusion, tumors were required to possess ≥80 percent tumor nuclei, but < 50% necrosis (to ensure the viability of tissues), and were otherwise trimmed or sliced to meet these specifications.

In 35 patient samples analyzed at MDA, a PCR-based primer extension assay was used to screen for mutations in frequently reported hot spots. This was performed using PCR, detecting up to 11 genes, or a next generation sequencing (NGS)-based analysis, for the detection of a total of 46 genes, as performed on the DNA extracted from the sample in a CLIA-certified molecular diagnostics laboratory. Details of the mutations detected in individual genes were reported. A minimum of 250x coverage is required at a given base for the interpretation of a wild-type or variant call. Although the NGS platform is capable of achieving a much higher analytical sensitivity, for clinical purposes, we determined the effective lower limit of detection of this assay (analytical sensitivity) to be in the range of 5% (one mutant allele in the background of nineteen wild-type alleles) to 10% (one mutant allele in the background of nine wild-type alleles) by taking into consideration the depth of coverage at a given base and the ability to confirm low level mutations using independent conventional platforms. We required that the tumor nuclei represent 20% of the nuclei in the tested sample to avoid false negative results. Alternatively, in seven patient samples, immunohistochemistry markers were examined microscopically in tissue sections to determine the presence of mutant genes, including *IDH1*.

### Statistical methods and survival correlation

Patient condition was correlated to a variety of tumor metrics derived from diffusion imaging or clinical parameters employing scatter plots with linear regression lines, univariate, and/or multivariate analysis methods (selected results are shown). Metrics tested include tract-length or volume of the tumor, derived from the whole tumor, tumor-core, tumor-shell, or normal brain. The most effective method for the analysis of tumor sub-regions was the application of tract-length filters (1-20 mm for core and 25-55 mm for shell). Other methods (e.g. Gaussian mixture model) were limited by inherent bias for short tracts in the whole tumor (1-55 mm) during tract generation. The average tract-lengths, and the composite volumes of tracts, were determined in DSI Studio under the specified length constraints. A cut-off of 50% was applied, and the Students T-test was used to evaluate high-versus-low values in these various metrics, with p value < 0.05 demonstrating significance. In addition to those listed above, additional metrics tested for high and low cut-off statistical differences were patient age, KPS, treatment applied (number of times a surgery or radiation was applied), and tumor genotype (number of patients with or without mutation). A common mutation was *IDH*, detected in six patients (MDA dataset), thus relevant *IDH* mutations were ruled out as a possible co-variable for tumor architecture. Further tumor genotype statistical comparisons were also examined. For further analysis, c/s ratio was chosen, given the greatest correlation (R^2^ value and slope of the regression line from low survival to high) compared to all other metrics identified. Co-variable analysis used the c/s ratio and the students T-test, with high or low c/s ratio as the ranking method for statistical comparisons. Kaplan-Meier estimation, the Log-rank test, and determination of hazard ratio were achieved using MATLAB software and KMPLOT (G. Cardillo, 2008) with confidence intervals (CI) and median survival times (Supplemental Figure S5), verified with manual estimation in Microsoft Excel software and with the students T-test. For comparison of TCGA/TCIA with MDA data, the former c/s ratio was normalized to the mean value of the later, and patients with less than 1-week survival time were excluded. Analysis completed using the statistical package R included univariate analysis to determine whether c/s ratio, age, and KPS (as continuous variables), or *IDH* mutation (as dichotomous variable of + or -) associate with survival. Multivariate analysis incorporating age and c/s ratio, or KPS and c/s ratio (both as continuous variables without arbitrary dichotomization) was conducted to determine if the variables independently associate with survival. Correlations between age and c/s ratio, or KPS and c/s ratio (as continuous variables) were determined.

## SUPPLEMENTARY MATERIALS FIGURES AND TABLES




