# Supplementary material for: Association between tumor architecture derived from generalized Q-space MRI and survival in glioblastoma
Source: Oncotarget. 2017 Mar 16;8(26):41815–26. doi: 10.18632/oncotarget.16296 (PMC5522030; doi:10.18632/oncotarget.16296)
Supplement: Supplementary file 1 [file oncotarget-08-41815-s001.pdf]

Association between tumor architecture derived from generalized Q-space MRI and survival in glioblastoma

Supplementary Material

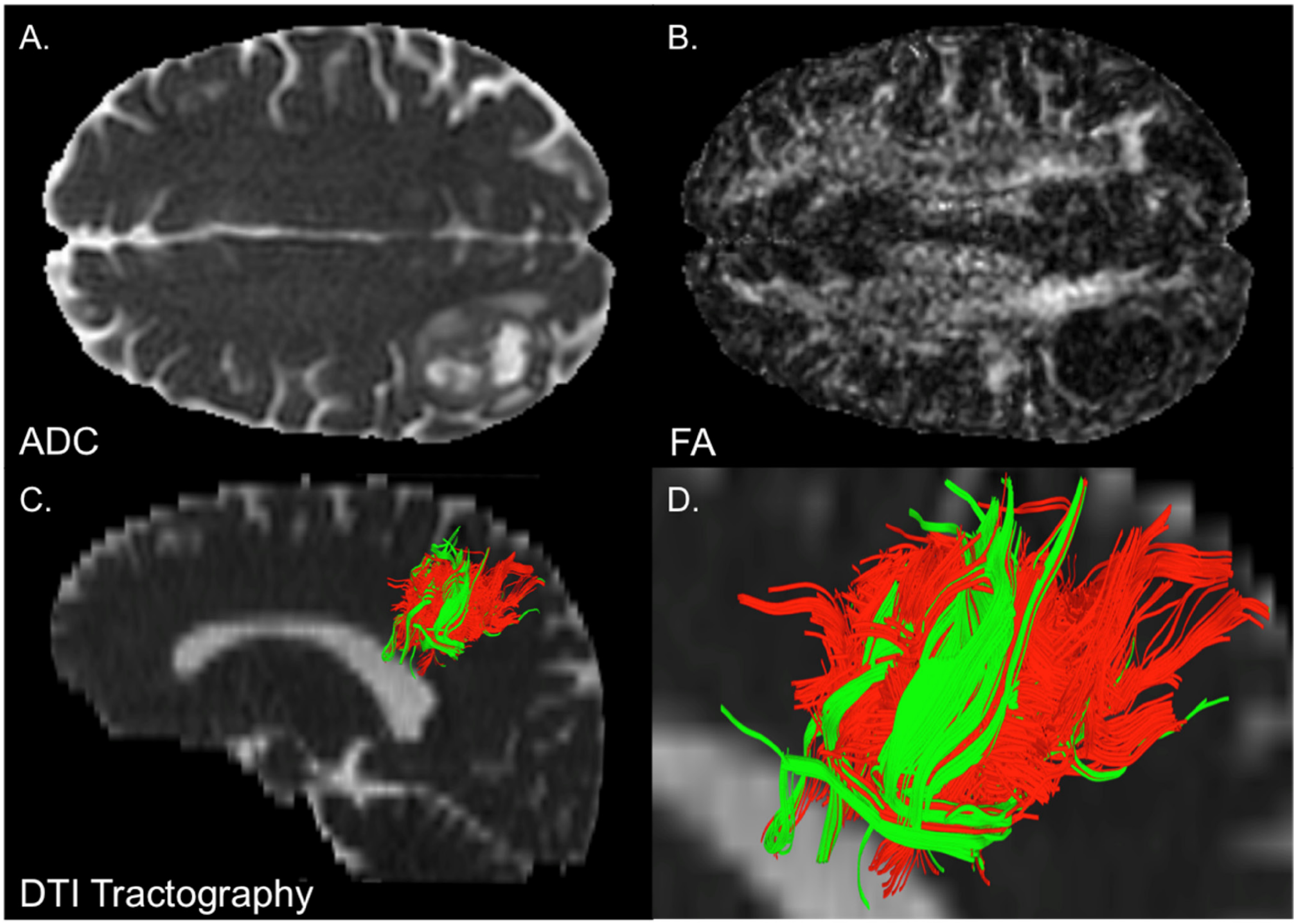

Supplementary Figure S1: Diffusion tensor imaging (DTI) analysis methods in patients with glioblastoma do not display regional heterogeniety. Representative patient from the University of Texas, MD Anderson Cancer Center (MDA) #1 with glioblastoma, demonstrating clear tumor borders, as scanned employing a diffusion-weighted MRI (DW-MRI; axial view) pulse sequence with analysis by apparent diffusion coefficient (ADC in A) and fractional anisotropy (FA in B). DTI with tractography for the analysis of short (1-20 mm) and long (25-55 mm) tract-length in glioblastoma is shown (in C and D) with limited distinction between core and shell, as compared with GQI analysis (patient MDA #1 is shown in the main text, Fig. 4, A-D).

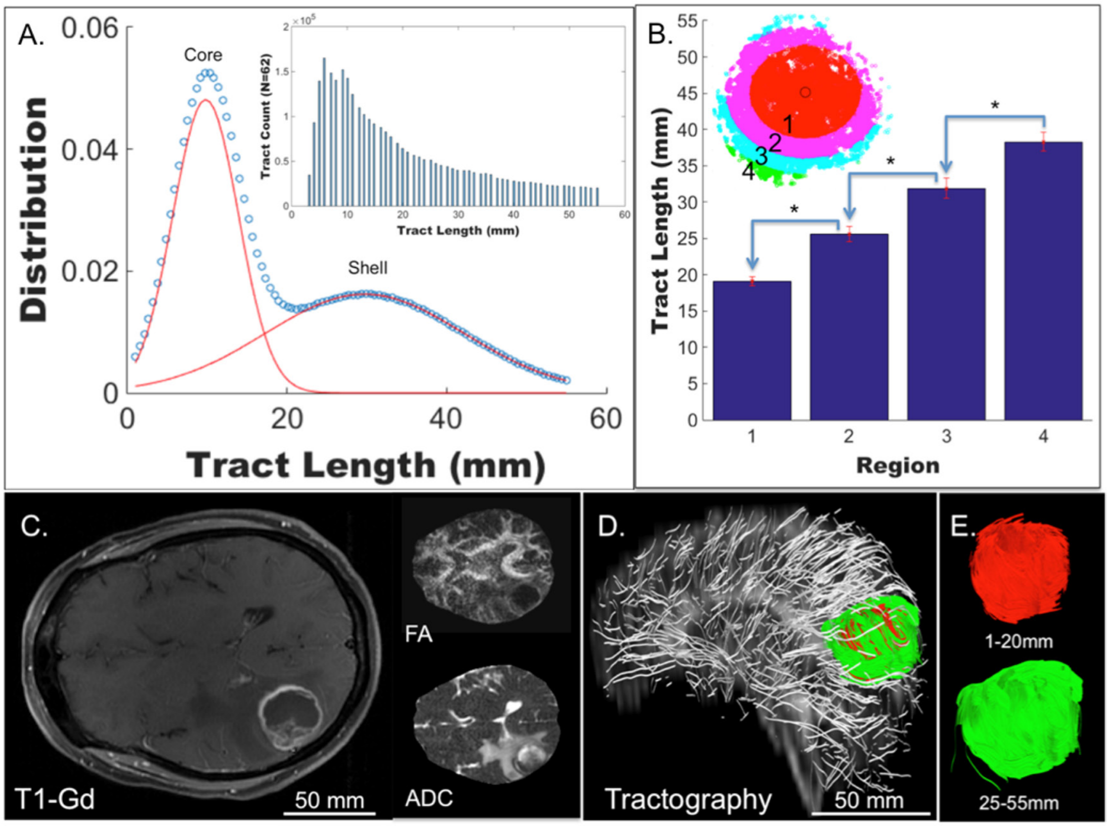

Supplementary Figure S2: Glioblastoma tumor architecture determined from GQI tractography in the MDA patient dataset (N=62). A. Two statistically distinct aligned cellular populations were demonstrated, with a bi-Gaussian distribution of tract-length in 62 patients, mean tract-lengths of 9.7980 mm and 29.7343 mm, and with mixing proportions of 0.496929 and 0.503071, respectively. B. The same 62 patients with quantification of tract-length at spatially distinct concentric regions grouped radially from the axial-orientation at the tumor center, defined from the average of tract points, to the edge of the tumors, consecutively labeled into four regions ($P<0.005$ for each compared region). Representative patient MDA #2 with glioblastoma scanned employing conventional MRI (in C) and analyzed with GQI for tumor architecture (D; expanded in E) demonstrating high core-shell diffusion tract-length ratio (c/s ratio). Tract-length filters were applied, with the core shown in red (1-20 mm tracts) and the shell in green (25-55 mm tracts). Substantial overlap of the two regions was observed in this patient, and was representative of the difference between the high and low c/s ratio patients. The tumor architecture can be compared between the patients; low c/s ratio (patient survival time 155 days) is shown in main text, Fig. 4 A-D, and a high c/s ratio (patient survival time 1031 days) is shown here in C-E.

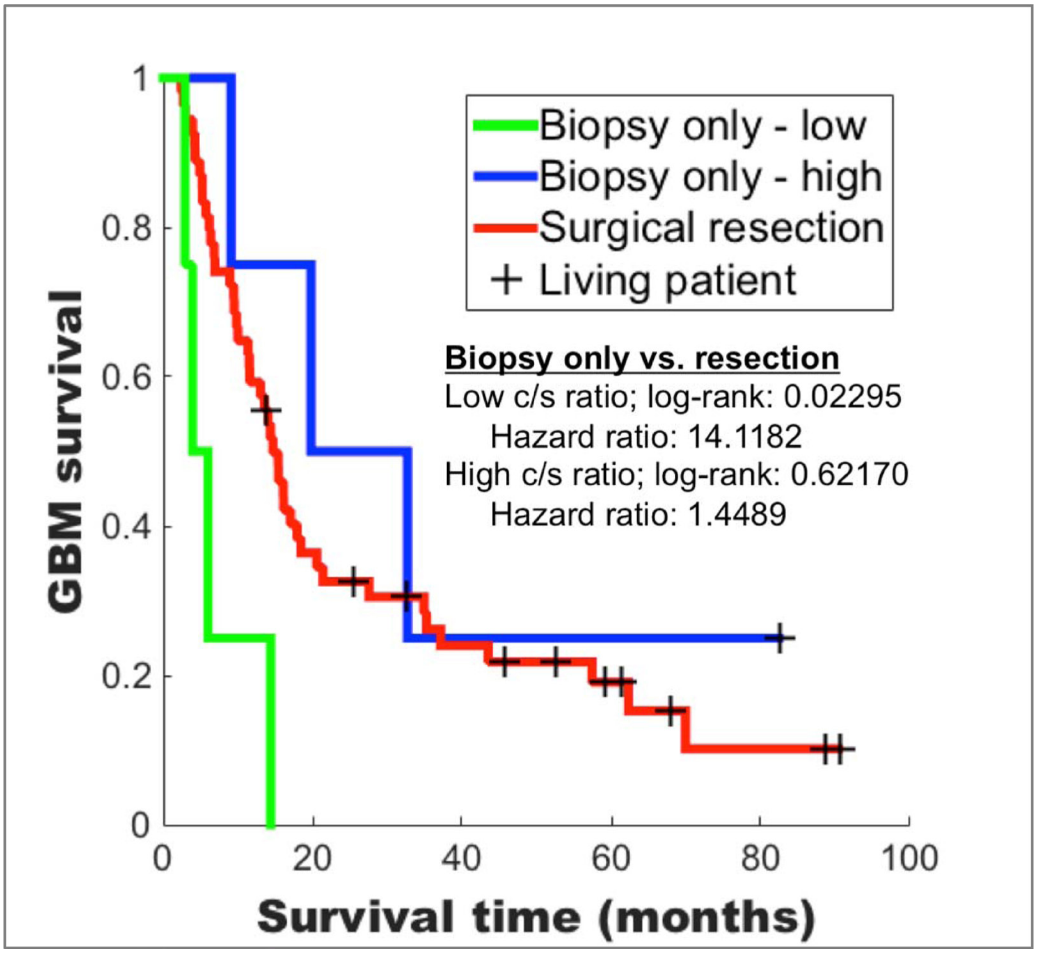

Supplementary Figure S3: Survival correlation using core-shell diffusion tract-length ratio (c/s ratio) in patients receiving biopsy only compared to all patients receiving surgical resection. The combination of radiation therapy and chemotherapy without resection (biopsy only) yielded a significantly worse outcome in low c/s ratio patients, while in high c/s ratio patients, radiation therapy and chemotherapy resulted in statistically similar outcome to surgical resection with combination therapy in all patients. Eight patients out of 62 in the MDA cohort received biopsy only, and were then treated with a combination of chemotherapy and radiation. When compared to all patients receiving resection, a significantly worse prognosis of p value = 0.02295 by the log-rank test (N=4; hazard ratio of 14.1182) was found in low c/s ratio patients, but not high c/s ratio patients (p value = 0.62170; N=4; hazard ratio of 1.4489). The remaining patients (N=54) were treated with surgical resection plus radiation (3 patients), resection plus chemotherapy (1 patient), or resection plus both (50 patients).

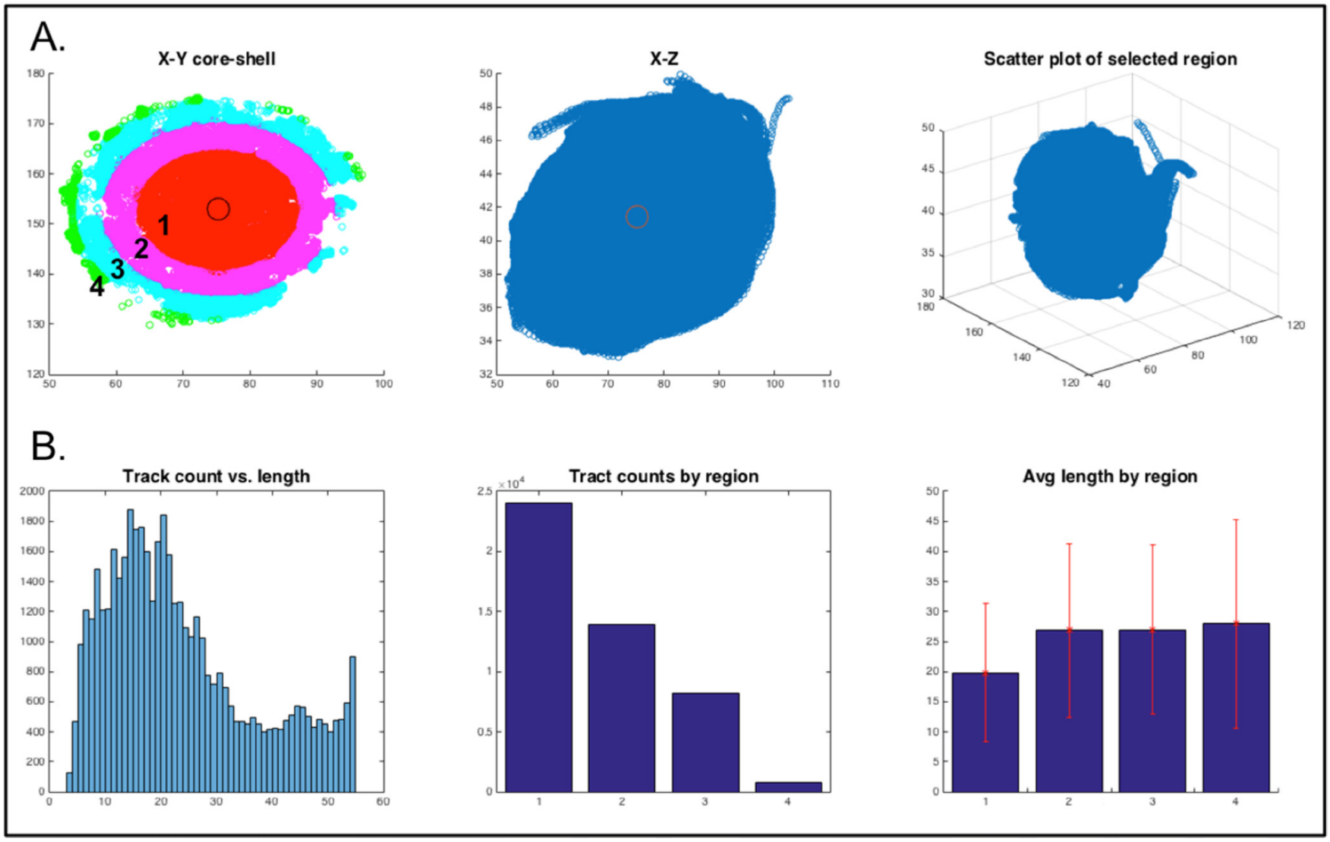

Supplementary Figure S4: Sample patient-specific output of the tract analysis code written in MATLAB from representative patient TCGA-06-5412. Analysis was carried out on an individual basis for both the TCGA (N=24) and the MDA (N-62) patient data sets, followed by data aggregation for each cohort. A. The center point of an individual tumor was determined from ROI specific patient tumor tractography files (1-55 mm tract-length and a 1 mm step size) exported from DSI studio software. The tumor was radially sliced into four equivalent areas consecutively labeled from the tumor center with 20% sampling in the axial orientation. Labels represent distance in mm for X-Y, X-Z, and X-Y-Z axes, respectively from left to right. B. Tract analysis carried out included tract count versus tract-length (left panel; distance in mm); tract counts by region (middle panel; regions are consecutively labeled from the tumor center); and average tract-length by region (right panel; +/- standard error of the mean, SEM). See Supplemental MATLAB code or https://github.com/eriktaylor/ for more details.

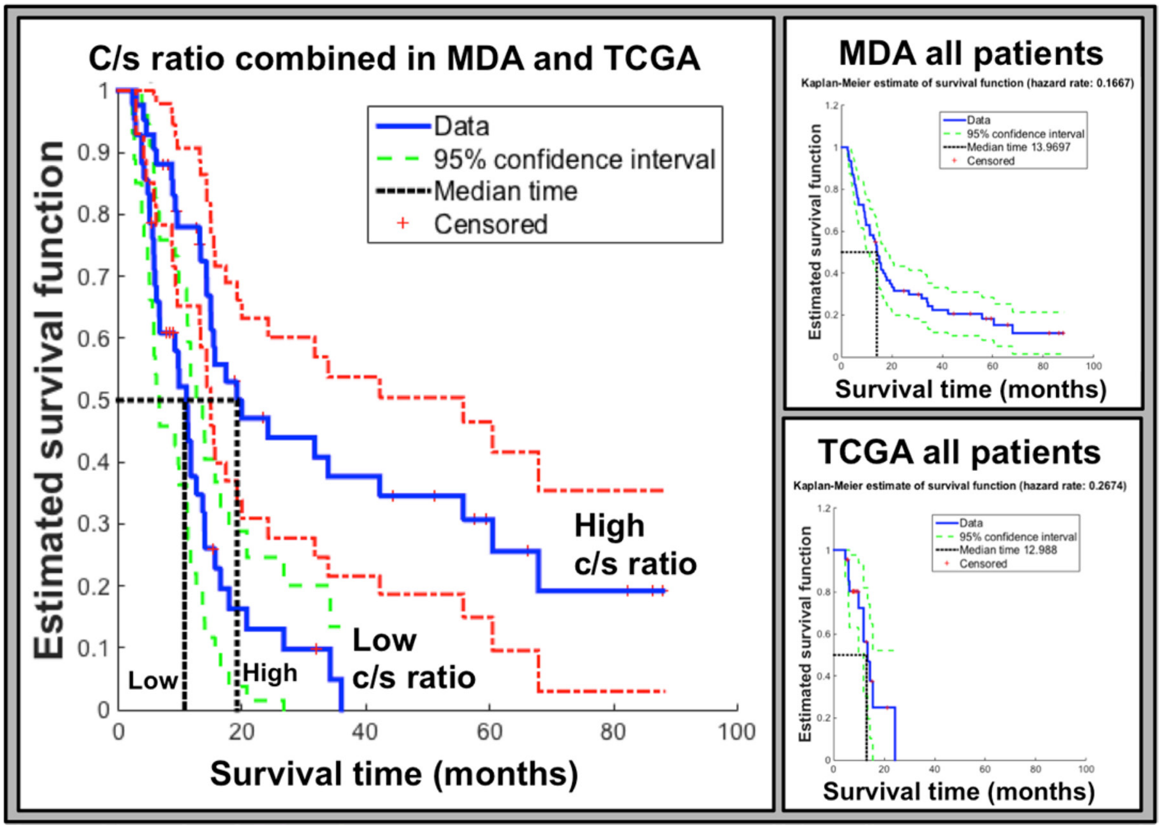

Supplementary Figure S5: MDA and TCGA Kaplan-Meier survival curves with confidence intervals (CI) and median survival times. Glioblastoma patient survival versus GQI derived core-shell diffusion tract-length ratio (c/s ratio) normalized to the mean across all patients (MDA plus TCGA; N=86; left panel). A significant difference between the high and low c/s ratio populations in the combined MDA and TCGA dataset was found, at 50% threshold, with a p value = 0.00004 by the log-rank test and the hazard ratio between the groups was calculated to be 3.3117. Median survival times for the patients in the combined c/s ratio dataset was 19.2 months for high c/s ratio, and was 10.7 months for the low c/s ratio patients. Inset right, survival in the TCGA and MDA datasets were statistically equivalent, with p value = 0.70644 by the log-rank test with confidence intervals and median survival times shown (13.9697 months for all MDA patients and 12.988 months for all TCGA patients).
