# Supplementary material for: Association between tumor architecture derived from generalized Q-space MRI and survival in glioblastoma
Source: Oncotarget. 2017 Mar 16;8(26):41815–26. doi: 10.18632/oncotarget.16296 (PMC5522030; doi:10.18632/oncotarget.16296)
Supplement: Supplementary file 2 [file oncotarget-08-41815-s002.docx]

**%%Supplemental MATLAB code%%**

%endpoint_plot_script

%Input - track file from diffusion spectrum imaging studio

%(DSI Studio; http://dsi-studio.labsolver.org)

%Use export to text (.txt) file

STEPSIZE = 1; %Human

%STEPSIZE = 0.1; %RAT

%ZCOVERAGE = 0.05; %Percent Z-slice (axial) coverage

ZCOVERAGE = 0.20;

%For swapping X, Y, and Z coordinates

xloc=1;

yloc=2;

zloc=3;

% File Open

%Files must end with .txt to be located in the tract-file directory

locationoffiles='/Users/erikntaylor/Desktop/Cancer_study_Houston/TCIA_GBM/TCGA_Histogram_Validation/';

filesindirectory = dir(strcat(locationoffiles, '*.txt'));

numfids = length(filesindirectory);

numfids=3; %required to testing fewer # of files (three in this case)

loopcount=1;

barmeanST=zeros(numfids,4);

barstdST=zeros(numfids,4);

%Create a location to save the output figures

savefile='/Users/erikntaylor/Desktop/Cancer_study_Houston/TCIA_GBM/TCGA_Histogram_Validation/FIGURES/';

%for loopcount=1:numfids %all files

for loopcount=1:3 %First three files

fName = strcat(locationoffiles,filesindirectory(loopcount).name);

fid = fopen(fName); %# Open the file

mat1=dlmread(fName);

fclose(fid);

%%%%%%%%%%%%%%%%%%%%%%%%%%%%%%%

% step size must be 1mm

mats=mat1; %for storing information to mesh

mat1(mat1 == 0) = NaN;

%%%%%%%%%%%%%%%%%%%%%%%%%%%%%%%

% Track Size

j=size(mat1,1); %number of tracks

k=(size(mat1,2)/3); %length of track

Storage=NaN(j,1);

mat1REDEF=NaN(j,size(mat1,2));

Vol=transpose(reshape(transpose(mat1), 3, (size(mat1, 1)*size(mat1, 2))/3));

X=Vol(:,xloc);

Y=Vol(:,yloc);

Z=Vol(:,zloc);

Xtract=transpose(reshape(X, k, j));

Ytract=transpose(reshape(Y, k, j));

Ztract=transpose(reshape(Z, k, j));

%%%%%%%%%%%%%%%%%%%%%%

%FIND CENTER OF TUMOR AND PLOT

meanX=mean(X, 'omitnan');

meanY=mean(Y, 'omitnan');

meanZ=mean(Z, 'omitnan');

figure;

subplot(2,3,2)%%%%%%%%%%%%%%%%%%%%%%%%X-Z

scatter(X,Z); hold on;

plot(meanX,meanZ, 'o', 'MarkerSize',20)

title('X-Z'); hold off;

%%%%%%%%%%%%%%%%%%%%%%

%Define range off Z

interval=(max(Z)-min(Z))*ZCOVERAGE;

%interval=(max(Z)-min(Z))/2*sqrt(1/4); %use same definition as below, i.e. D/2*sqrt(1/4)

uprange=meanZ+interval;

lowrange=meanZ-interval;

%%%%%%%%%%%%%%%%%%%%%%

% length of a particular vector

i=1;

jump=0;

for i=1:j

if (find(Ztract(i,:)>lowrange & Ztract(i,:)<uprange))>0

jump=jump+1;

mat1REDEF(jump,:)=mat1(i,:);

NumberofNAN = (sum(isnan(mat1(i,:))));

LengthofNAN = k - NumberofNAN/3; %length of a particular track

Storage(jump,1)=LengthofNAN*STEPSIZE; %Stores a particular tumor file

end

end

subplot(2,3,4)

histogram(Storage);

title('Track count vs. length');

%%%%%%%%%%%%%%%%%%%%%%

%Plot range of Z

mat2REDEF=mat1REDEF(1:jump,:);

StorageREDEF=Storage(1:jump,:);

VolR=transpose(reshape(transpose(mat2REDEF), 3, (size(mat2REDEF, 1)*size(mat2REDEF, 2))/3));

xsel=VolR(:,xloc);

ysel=VolR(:,yloc);

zsel=VolR(:,zloc);

xtractsel=transpose(reshape(xsel, k, jump));

ytractsel=transpose(reshape(ysel, k, jump));

ztractsel=transpose(reshape(zsel, k, jump));

subplot(2,3,3)

h=scatter3(xsel, ysel, zsel);

title('Scatter plot of selected region');

%Define core and shell from selected points

%rx=(max(xsel)-mean(xsel)); %x-axis radius of tumor

%tdistx=abs(xsel(i)-mean(xsel)) %dist in tumor < radius of tumor

%sqrt((tdistx)^2+(tdisty)^2) %any location in tumor <radius of tumor

%Define radius of equal portions, as: ri=R*sqrt(i/n);

%Thus, ri=R*sqrt(i/4)

Core1=NaN(jump,size(mat1,2));

Core2=NaN(jump,size(mat1,2));

Core3=NaN(jump,size(mat1,2));

Core4=NaN(jump,size(mat1,2));

Corestore1=NaN(jump,1);

Corestore2=NaN(jump,1);

Corestore3=NaN(jump,1);

Corestore4=NaN(jump,1);

corecount1=0;

corecount2=0;

corecount3=0;

corecount4=0;

midpx=mean(xsel, 'omitNan');

midpy=mean(ysel, 'omitNan');

rx=(max(xsel)-min(xsel))/2;

ry=(max(ysel)-min(ysel))/2;

if rx>ry %define as max radius (biggest circle)

radius=rx;

else

radius=ry;

end

for i=1:jump

if (find(sqrt((abs(xtractsel(i)-midpx))^2+(ytractsel(i)-midpy)^2)<(radius*sqrt(1/4))));

corecount1=corecount1+1;

Core1(corecount1,:)=mat2REDEF(i,:);

Corestore1(corecount1)=StorageREDEF(i);

elseif (find(sqrt((abs(xtractsel(i)-midpx))^2+(ytractsel(i)-midpy)^2)<(radius*sqrt(2/4))));

corecount2=corecount2+1;

Core2(corecount2,:)=mat2REDEF(i,:);

Corestore2(corecount2)=StorageREDEF(i);

elseif (find(sqrt((abs(xtractsel(i)-midpx))^2+(ytractsel(i)-midpy)^2)<(radius*sqrt(3/4))));

corecount3=corecount3+1;

Core3(corecount3,:)=mat2REDEF(i,:);

Corestore3(corecount3)=StorageREDEF(i);

else

corecount4=corecount4+1;

Core4(corecount4,:)=mat2REDEF(i,:);

Corestore4(corecount4)=StorageREDEF(i);

end

end

subplot(2,3,1)

scatter(Core1(:,xloc), Core1(:,yloc), 'r')

hold on;

scatter(Core2(:,xloc), Core2(:,yloc), 'm')

scatter(Core3(:,xloc), Core3(:,yloc), 'c')

scatter(Core4(:,xloc), Core4(:,yloc), 'g')

plot(meanX,meanY, 'o', 'MarkerSize',20, 'MarkerEdgeColor','k')

title('X-Y core-shell');hold off;

subplot(2,3,5)

bar(1:4,[corecount1 corecount2 corecount3 corecount4]);

title('Tract counts by region');

subplot(2,3,6)

barmean=[0 0 0 0];

barstd=[0 0 0 0];

barmean(1)=mean(Corestore1, 'omitnan');

barstd(1)=std(Corestore1,'omitnan');

barmean(2)=mean(Corestore2, 'omitnan');

barstd(2)=std(Corestore2,'omitnan');

barmean(3)=mean(Corestore3, 'omitnan');

barstd(3)=std(Corestore3,'omitnan');

barmean(4)=mean(Corestore4, 'omitnan');

barstd(4)=std(Corestore4,'omitnan');

title('Avg tract length by region (i.e. red core=1, green shell =4');

hold on

bar(1:4,barmean); %Step size 1 (human)

errorbar(1:4,barmean,barstd, 'rx');

hold off

%Store data for each patient as required

%loopcount

barmeanST(loopcount, :)=barmean(1, :);

barstdST(loopcount,:)=barstd(1, :);

SAVEHERE=strcat(savefile,filesindirectory(loopcount).name);

savefig(strcat(SAVEHERE, '*.fig'))

close all

clear Core1

clear Core2

clear Core3

clear Core4

clear mat1

clear mat2REDEF

clear mat1REDEF

clear mats

clear Storage

clear StorageREDEF

clear Vol

clear VolR

clear X

clear xsel

clear Xtract

clear xtractsel

clear Y

clear ysel

clear Ytract

clear ytractsel

clear Z

clear zsel

clear Ztract

clear ztractsel

end

barmeanF=mean(barmeanST, 'omitnan');

%Sum variance, average, and take the square root (stdev)

barstdF=std(barmeanST, 'omitnan')/sqrt(numfids);

figure

hold on

bar(1:4,barmeanF)

errorbar(1:4,barmeanF,barstdF, 'rx')

hold off

axis([0.5 4.5 0 55]); %for human

%axis([0.5 4.5 0 15]); %For rat

set(gca,'XTick',[1 2 3 4]);

set(gca,'FontSize',25, 'FontName', 'Arial');

xlhand = get(gca,'xlabel');

set(xlhand,'string','Region','fontsize',30, 'FontName', 'Arial Black');

ylhand = get(gca,'ylabel');

set(ylhand,'string','Tract Length (mm)','fontsize',30, 'FontName', 'Arial Black');

%Used for the rat to display two groups instead of 4

figure

barmeanF2=[sum(barmeanF(1:2))/2 sum(barmeanF(3:4))/2];

%Sum variance, average, and take the square root (stdev)

barstdF2=sqrt([sum(barstdF(1:2).^2)/2 sum(barstdF(1:2).^2)/2]);

hold on;

bar(1:2,barmeanF2)

errorbar(1:2,barmeanF2,barstdF2, 'rx')

hold off
